# CD161 expression characterizes a subpopulation of human regulatory T cells that produces IL-17 in a STAT3-dependent manner

**DOI:** 10.1002/eji.201243296

**Published:** 2013-05-15

**Authors:** Behdad Afzali, Peter J Mitchell, Francis C Edozie, Giovanni AM Povoleri, Sophie E Dowson, Laura Demandt, Gina Walter, James B Canavan, Cristiano Scotta, Bina Menon, Prabhjoat S Chana, Wafa Khamri, Shahram Y Kordasti, Susanne Heck, Bodo Grimbacher, Timothy Tree, Andrew P Cope, Leonie S Taams, Robert I Lechler, Susan John, Giovanna Lombardi

**Affiliations:** 1 Medical Research Council Centre for Transplantation, King's College London, King's Health Partners, Guy's HospitalLondon, UK; 2 Centre for Molecular and Cellular Biology of Inflammation (CMCBI), King's College London, King's Health Partners, Guy's HospitalLondon, UK; 3 Department of Immunobiology, King's College London, King's Health Partners, Guy's HospitalLondon, UK; 4 Department of Hematological Medicine, King's College London, King's Health Partners, Guy's HospitalLondon, UK; 5 Academic Department of Rheumatology, King's College London, King's Health Partners, Guy's HospitalLondon, UK; 6 Flow Cytometry Core Facility at the National Institute for Health Research Guy's and St Thomas’ NHS Foundation Trust/King's College London comprehensive Biomedical Research Centre, Guy's HospitalLondon, UK; 7 Department of Immunology and Molecular Pathology, Royal Free Hospital, University CollegeLondon, UK

**Keywords:** Conversion, Human, Regulatory T (Treg) cells, STAT3, Th17

## Abstract

Treg cells are critical for the prevention of autoimmune diseases and are thus prime candidates for cell-based clinical therapy. However, human Treg cells are “plastic”, and are able to produce IL-17 under inflammatory conditions. Here, we identify and characterize the human Treg subpopulation that can be induced to produce IL-17 and identify its mechanisms. We confirm that a subpopulation of human Treg cells produces IL-17 in vitro when activated in the presence of IL-1β, but not IL-6. “IL-17 potential” is restricted to population III (CD4^+^CD25^hi^CD127^lo^CD45RA^−^) Treg cells expressing the natural killer cell marker CD161. We show that these cells are functionally as suppressive and have similar phenotypic/molecular characteristics to other subpopulations of Treg cells and retain their suppressive function following IL-17 induction. Importantly, we find that IL-17 production is STAT3 dependent, with Treg cells from patients with STAT3 mutations unable to make IL-17. Finally, we show that CD161^+^ population III Treg cells accumulate in inflamed joints of patients with inflammatory arthritis and are the predominant IL-17-producing Treg-cell population at these sites. As IL-17 production from this Treg-cell subpopulation is not accompanied by a loss of regulatory function, in the context of cell therapy, exclusion of these cells from the cell product may not be necessary.

## Introduction

Physiological maintenance of peripheral tolerance is critically dependent on a population of circulating cells committed to suppressor function. Of these, CD4^+^ “regulatory” T (Treg) cells expressing high levels of the IL-2 receptor α chain (CD25) and the transcription factor FOXP3 are the most important [1]. Treg cells suppress the proliferation and effector function of CD4^+^CD25^−^ effector T (Teff) cells to polyclonal and antigen-specific stimuli [2]. Loss of the Treg-cell lineage, through mutations in the *FOXP3* gene, manifests in life-threatening X-linked autoimmune diseases in mammals (the Scurfy strain in mice [3] and human immunodysregulation, polyendocrinopathy, enteropathy, and X-linked (IPEX) syndrome [4]). In addition, functional deficits in Treg cells have been proposed to contribute to the development or severity of autoimmune diseases in man [5,6]. Conversely, administration of Treg cells in murine models controls experimental allergic [7] and autoimmune diseases [8] and can prevent rejection of allografts [9] while in borderline or acutely rejecting human renal and liver transplant specimens, Treg-cell numbers correlate positively with better outcomes [10,11]. These properties, together with the observation that human Treg cells can be expanded ex vivo, either polyclonally [12,13] or for a given specificity [14], make Treg cells ideal candidates for tolerance-inducing cell therapy in human autoimmune diseases and transplantation [15]. Indeed, small-scale trials have demonstrated beneficial outcomes in the prevention or treatment of postbone marrow transplantation human graft versus host disease [16].

Emerging concepts of mammalian CD4^+^ T-helper (Th) lineage commitment [17] suggest that Th-cell fate is not as irreversible as previously thought, and that lineage reprogramming can occur through the inducible expression of key transcription factors [17]. Differentiation of Treg cells from naïve murine precursors is reciprocally linked to that of the Th17-cell lineage through a common requirement for TGF-β with the presence or absence of IL-6 skewing differentiation toward Th17 or Treg cells, respectively [18]. Th17 cells express the transcription factors ROR-α and ROR-γt (RORA and RORC2 in humans) and produce the proinflammatory cytokine IL-17. The Th17-cell lineage is functionally nonredundant for the elimination of extracellular pathogens [19] and is a major pathogenic lineage in the development and/or activity of autoimmune diseases and organ rejection in humans [20]. However, Th17 cells generated with TGF-β and IL-6 demonstrate unstable lineage commitment and undergo fate switching to alternate lineages, in particular Th1, both in vitro and in vivo [21–23].

Likewise, Treg-cell lineage commitment has recently been questioned, with demonstrations that their regulatory function can be subverted in the context of infection [24] and that they can be induced to express the phenotypic profile of Th17 cells in the presence of inflammatory cytokines, namely IL-1 and IL-6 [25–27]. However, lineage reprogramming of Treg cells remains controversial, as fate-mapping studies in murine models have failed to replicate the plasticity data [28]. Nevertheless, “Th17 converted” Treg cells have been identified in inflamed, but not in noninflamed, bowel from patients with Crohn's disease in man [29]. It is unlikely that these data are the result of “outgrowth” of Foxp3^−^ non-Treg contaminants as these cells do not expand when co-transferred with Foxp3^+^ populations in the lymphopenic hosts [30]. Indeed, it was recently shown that specific human Treg-cell subpopulations can evolve that functionally mirror analogous effector Th-cell subsets, guided by proinflammatory cues during an immune response [31]. Thus, Treg-cell plasticity is of fundamental importance to understand the development of autoimmune diseases and anticipation of adverse effects in programs of Treg-cell-based therapy.

In this study, we confirm that human Treg cells in vitro produce IL-17 when activated in the presence of IL-1β, in a STAT3-dependent manner and that this function is the prerogative of a subpopulation of Treg cells within population III [32] expressing CD161. However, these cells maintain their suppressive function. Finally, we show that these Treg cells are enriched within the inflamed joints of patients with inflammatory arthritis relative to matched peripheral blood (PB).

## Results

### Human Treg cells produce IL-17 in vitro

**Figure 1:**
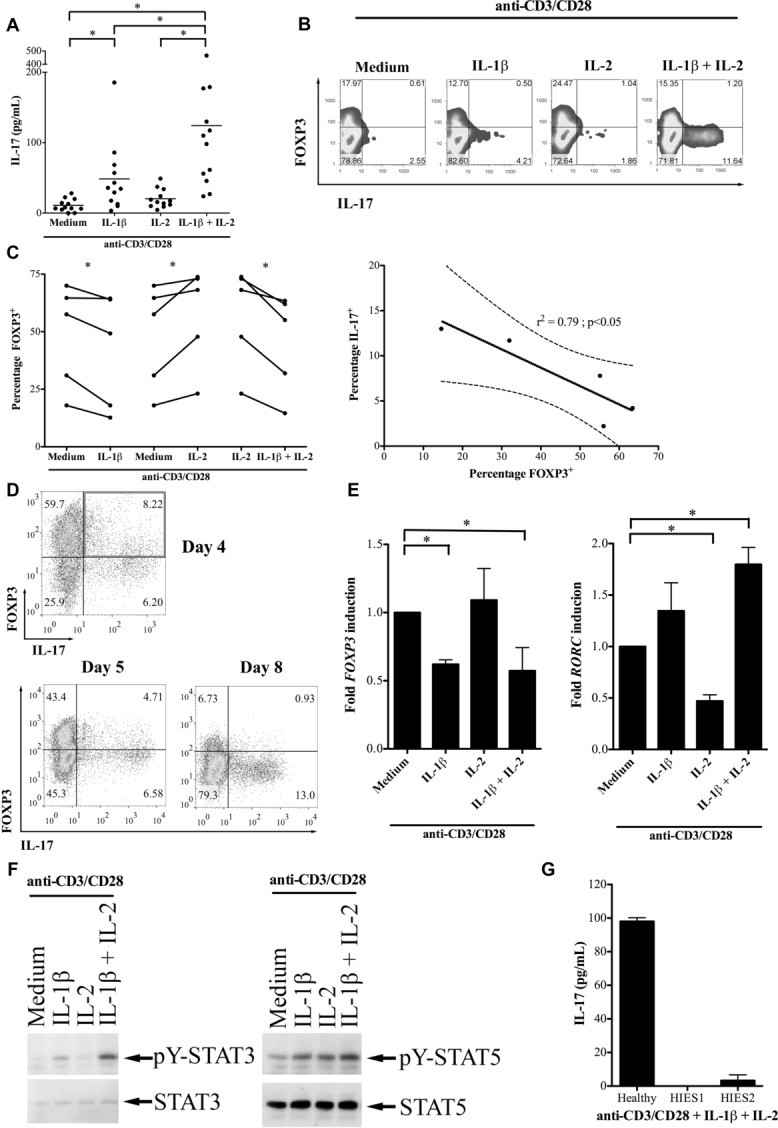
Human Treg cells are induced to produce IL-17 and to downregulate FOXP3 by IL-1β. (A) “Total” CD4^+^CD25^+^ Treg cells from healthy donors were activated with anti-CD3/CD28 in medium supplemented with IL-1β, IL-2 or IL-1β+IL-2. IL-17 production was measured by ELISA and shown as mean of 12 donors. (B) A representative example of ICS for IL-17 and FOXP3 in which Treg cells were activated for 5 days with anti-CD3/CD28 together with the cytokines indicated. Data shown are from one experiment representative of five performed. (C) Cumulative data from five independent experiments showing FOXP3 staining in Treg cells activated with anti-CD3/CD28 together with cytokines (left) and negative correlation between the percentages of FOXP3^+^ and IL-17^+^ cells (right; *r*^2^ = 0.79, *p* < 0.05). The solid and dashed lines show the linear regression and 95% confidence interval, respectively (right). (D) A representative ICS for FOXP3 and IL-17 expression in Treg cells activated with anti-CD3/CD28 in the presence of IL-1β and IL-2, highlighting the double-positive population on day 4 (top, upper right quadrant) plus a kinetic transition through a double-positive phase (bottom). Data shown are from one experiment representative of five performed. (E) qRT-PCR for *FOXP3* (left) and *RORC* (right) from Treg cells activated with anti-CD3/CD28 with and without cytokine. Data are normalized to unsupplemented medium; shown are mean +SD of triplicate samples and are from one experiment representative of two performed. (F) Representative Western blot showing total and phosphotylated (pY) STAT3 in Treg cells activated with anti-CD3/CD28 in the presence of the cytokines indicated. Data shown are from one experiment representative of four performed. (G) IL-17 production from Treg cells of a healthy control and two patients with hyper-IgE syndrome activated with anti-CD3/CD28 and exogenous cytokine. Data are shown as mean +SD and are from one experiment. *p < 0.05, One-way ANOVA (A), paired *t*-test (C, left panel), F test (C, right panel).

It has been shown both in murine models and in man that Treg cells are plastic. In order to identify the population of Treg cells responsible for the conversion to IL-17-producing cells and the mechanisms behind this event, freshly isolated bead-enriched “whole” human Treg cells (CD4^+^CD25^+^) were initially activated in vitro in the presence of IL-1β, as previously reported [25,26], and/or IL-2. We observed that Treg cells produced IL-17 when in the presence of IL-1β and the highest levels were found when IL-2 was further added to the culture (Fig. 1A), in part due to IL-2-mediated increased expression of IL-1R1 (Supporting Information Fig. 1), as previously shown [12]. The increased IL-17 mediated by IL-1β was confirmed with intracellular staining (ICS) and was accompanied by a reduction in FOXP3 (Fig. 1B and C). A negative correlation between the percentage of IL-17 and FOXP3 single-positive cells was observed following activation of Treg cells in the presence of IL-1β plus IL-2 (*r*^2^ = 0.79, *p* < 0.05) (Fig. 1C). The presence of IL-2, however, maintained a higher percentage of FOXP3^+^ cells in ex vivo activated Treg cells but the addition of IL-1β antagonized this effect (Fig. 1C). The conversion of Treg cells to Th17 cells, rather than outgrowth of a contaminating population, was suggested by repeated observation of cells that were double positive for both FOXP3 and IL-17 (Fig. 1D, top panel), plus a kinetic transition through a double-positive phase (Fig. 1D, lower panel and Supporting Information Fig. 2), indicative of a transitional stage. In the double-positive population, FOXP3 levels were lower in those cells expressing higher IL-17 levels (Fig. 1D).

Appropriate patterns of the transcription factors FOXP3 and RORC were identified in Treg cells treated with exogenous cytokine immediately after culture: IL-1β reduced *FOXP3* mRNA and elevated *RORC* transcripts, whereas IL-2 maintained high *FOXP3* but inhibited *RORC* transcription, as has been documented in mouse T cells [33]. The combination of the two cytokines showed dominant effects of IL-1β, characterized by quantitatively high *RORC* and low-*FOXP3* transcripts (Fig. 1E). However, the relatively small changes in transcription factor profiles observed (Fig. 1E) and the relatively low-frequency conversion seen with ICS (Fig. 1B) suggested that Th17 plasticity is a feature of some, but not all, cells contained in the “whole” human Treg-cell population (see below).

As naïve T-cell lineage commitment to Th17 is critically dependent on STAT3 activation [34], we assessed the status of STAT3-tyrosine phosphorylation of Treg cells activated in the presence of IL-1β, IL-2, or the combination of the two cytokines (Fig. 1F). As the earliest time point at which increased IL-17 could clearly be detected following IL-1β treatment of Treg-cell cultures was 3 days, cell extracts were prepared at this time point. STAT3 phosphorylation was significantly induced and sustained when Treg cells were activated in the presence of IL-1β or IL-1β+ IL-2 (Fig. 1F). In contrast, STAT5 tyrosine phosphorylation remained at similar levels in all conditions, as with TCR activation alone. Similar results were obtained from cells stimulated for 5 days (data not shown). These observations were corroborated by ICS for pY-STAT3 and pY-STAT5 in Treg cells (Supporting Information Fig. 3). Of note, and different from the mouse, IL-6 did not induce production of IL-17 by Treg cells (data not shown). Thus, additional molecular changes induced by IL-1β, but not IL-6, are required along with STAT3 activation for the expression of IL-17 by Treg cells.

Altogether, these findings indicate that increased RORC expression and pSTAT3 levels may compete with pSTAT5 to positively regulate the IL-17 locus and contribute to the increased IL-17 expression in IL-1β-treated Treg cells. To confirm the importance of STAT3 signaling in IL-1β-induced IL-17 production by Treg cells, Treg cells were separated from the PB of two patients with hyper-IgE syndrome (OMIM 147060). These subjects had dominant negative mutations in the SH2 domain of STAT3, impairing STAT3 activation [35]. Treg cells from these two patients and one healthy control were activated in culture medium containing IL-1β and IL-2, and supernatants tested 5 days later for IL-17 by ELISA. Although healthy donor Treg cells produced IL-17 in the presence of IL-1β and IL-2, Treg cells from STAT3 mutant patients produced negligible amounts (patient 1: 0 pg/mL; patient 2: 6 pg/mL) (Fig. 1G). Thus, the importance of STAT3 signaling for IL-17 production by Treg cells is firmly established as Treg cells from hyper-IgE syndrome patients with defective STAT3 proteins cannot differentiate along this pathway.

### A distinct subpopulation of human Treg cells undergoes IL-17 induction in vitro

**Figure 2:**
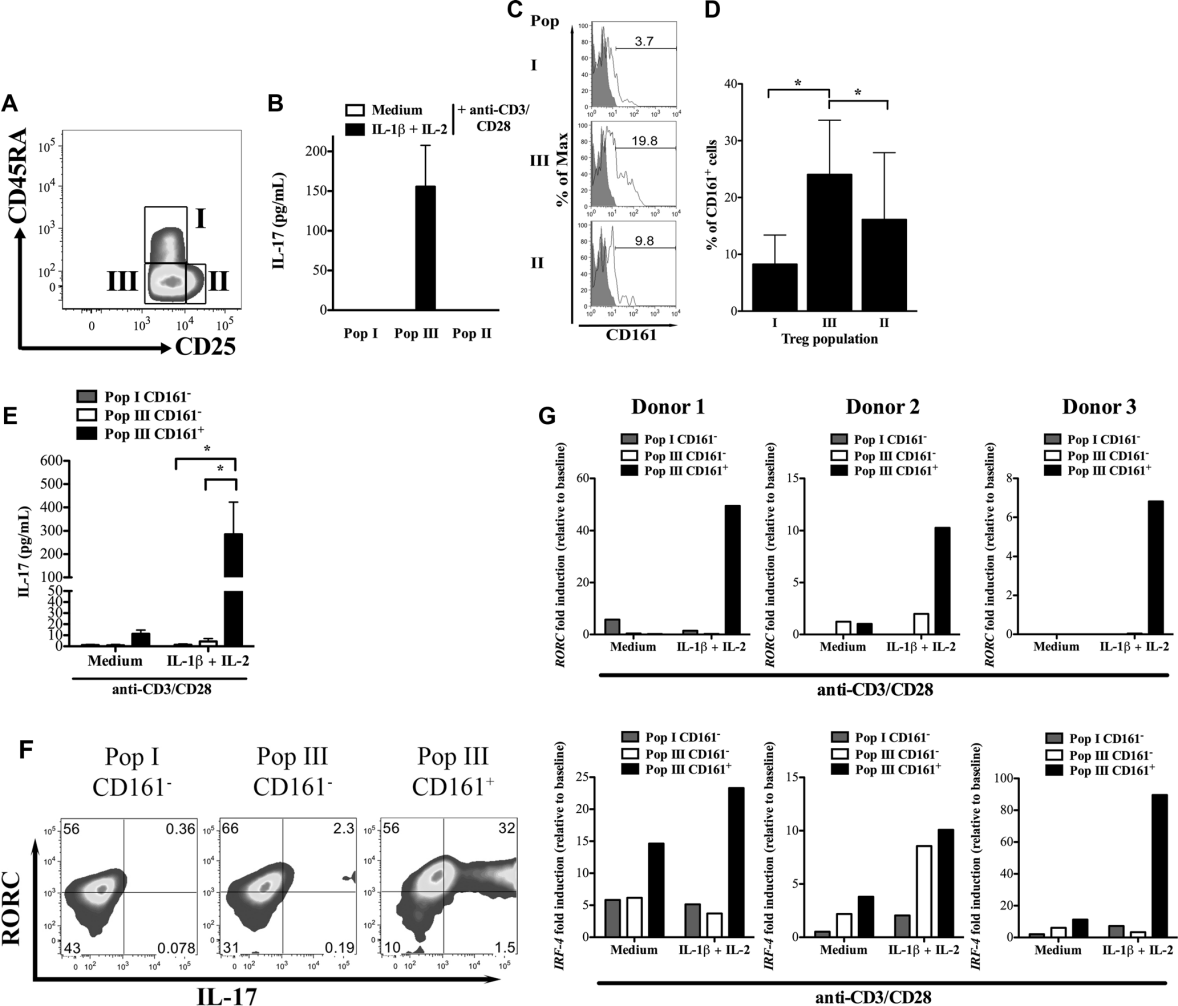
Population III CD161+ Treg cells are the population with “IL-17 potential”. (A) The gating strategy, adapted from Miyara et al. [32], used to identify populations I, II, and III CD4^+^CD25^hi^CD127^lo^ Treg cells is shown. A representative example from multiple experiments is shown (please see Supporting Information Fig. 4 for full gating pathway). (B) The IL-17 concentrations in supernatants of populations I, III, and II Treg cells activated with anti-CD3/CD28 coated beads in the presence of medium only (medium) or medium supplemented with IL-1β and IL-2 (IL-1β+IL-2) for 5 days were determined by ELISA. Data are shown as mean + SD pooled from three experiments using blood from three independent healthy donors. (C, D) CD161 expression on populations I, III, and II Treg cells was determined by flow cytometry, (C) a representative example and (D) the mean + SD of five donors is shown. (E) IL-17 production by population I CD161^−^, population III CD161^−^, and population III CD161^+^ Treg cells activated with anti-CD3/CD28 coated beads in the presence of medium alone (medium) or medium supplemented with IL-1β and IL-2 (IL-1β+IL-2) for 5 days are shown as mean +SD of data pooled from five experiments performed * *p* < 0.05, paired *t*-test. (F) RORγt and IL-17 expression in population I CD161^−^, population III CD161^−^, and population III CD161^+^ Treg cells after 5 days activation with anti-CD3/CD28 coated beads and IL-1β+IL-2 were determined by flow cytometry. Values in the gates are percentages. Data shown are representative of two independent experiments performed. (G) qRT-PCR showing fold induction of *RORC* (top) and *IRF-4* (bottom) in population I CD161^−^, population III CD161^−^, and population III CD161^+^ Treg cells activated with anti-CD3/CD28 coated beads in the presence of medium alone (medium) or medium supplemented with IL-1β and IL-2 (IL-1β+IL-2) for 5 days. The panels show individual data from three independent healthy donors.

Given the relatively small changes in transcription factor profiles observed (Fig. 1E) and the relatively low frequency of conversion seen with ICS (Fig. 1B) in “whole” bead-enriched CD4^+^CD25^+^ Treg cells, we hypothesized that IL-17 potential is restricted to one or more subpopulations of human Treg cells. Treg cells were FACS sorted into three subpopulations based on the expression of FOXP3 and CD45RA, namely CD4^+^CD25^++^CD127^lo^CD45RA^+^ (population I), CD4^+^CD25^+++^CD127^lo^CD45RA^−^ (population II), and CD4^+^CD25^++^CD127^lo^CD45RA^−^ (population III), as recently published [32] (Fig. 2A and Supporting Information Fig. 4). Following stimulation in vitro in the presence of IL-1β and IL-1β+IL-2, only population III was found to produce IL-17 (Fig. 2B).

To further refine the converting subpopulation of Treg cells, we tried to identify additional markers that can characterize it. As the NK-cell marker CD161 was shown by others to identify Th17 precursors among CD4^+^ human cells [36], its expression on populations I, II, and III was evaluated and was clearly more prevalent on Treg cells in population III than in populations I and II (Fig. 2C and D). Treg cells expressing CD161 (population III CD161^+^) and their CD161^−^ counterparts (population III CD161^−^) were FACS sorted, together with CD161^−^ cells from population I (population I CD161^−^) and in vitro Th17 conversion assessed as before. There were insufficient cells for cell sorting of CD161^+^ Treg cells from populations I and II to be included here. As shown in Figure 2E, only population III CD161^+^ cells produced IL-17 under IL-17-inducing conditions and did not convert in the absence of inflammatory signals. This latter point is important as it demonstrates that the now IL-17-producing population III CD161^+^ cells are not derived from previously committed, contaminating, Th17 cells.

To determine the frequency of IL-17-producing Treg cells, we performed ICS for RORC and IL-17 in the cell sorted populations after culture with anti-CD3/CD28/IL-1β/IL-2. A significant proportion of population III CD161^+^ cells produced IL-17, with the majority of cells also expressing RORC (Fig. 2F). This was not the case for population I or III CD161^−^ Treg cells (Fig. 2F). The induction of *RORC* and *Interferon-regulatory factor 4* (*IRF-4*), a critical transcription factor in Th17 differentiation [37], in these populations was confirmed by qRT-PCR, showing clearly a large upregulation of *RORC* in population III CD161^+^ activated under IL-17-inducing conditions (Fig. 2G, upper panels) and a similar induction of *IRF-4* (Fig. 2G, lower panels), suggesting that we have correctly identified a subset of Treg cells with IL-17 potential. In these cells, we observed a similar activation of STAT3 with IL-1β as before (data not shown).

Others have described CCR6 as a marker of Treg cells capable of producing IL-17 [25,31,36]. However, when we stained for CCR6 and IL-1R1 on population III Treg cells, we did not identify a significant difference between population III CD161^+^ and CD161^−^ cells in expression of CCR6 nor IL-1R1 (Supporting Information Fig. 5). Therefore, we concluded that CD161 is the marker that identifies Treg cells capable of being induced to produce IL-17 and that this property is not related to higher baseline expression of the IL-1R.

### Population III CD161^+^ Treg cells express less FOXP3 but are as suppressive as other Treg cells

**Figure 3:**
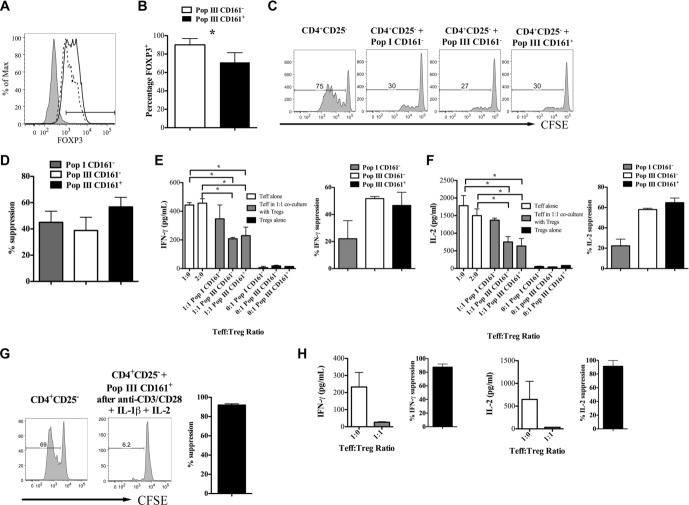
Population III CD161+ Treg cells express less FOXP3 but are as suppressive as other Treg-cell subpopulations. (A) A representative histogram of FOXP3 expression in freshly isolated Treg-cell populations III CD161^−^ (solid line) and III CD161^+^ (dashed line) relative to isotype control (shaded) is shown. (B) The percentage of FOXP3^+^ cells is also shown as mean + SD of four independent donors * < 0.05, paired *t*-test. (C, D) The suppressive effects of freshly isolated population I CD161^−^, population III CD161^−^, and population III CD161^+^ cells on proliferation of autologous CFSE-labeled Teff cells are shown as (C) a representative flow cytometry example and (D) mean + SD of five samples pooled from five independent experiments. (E, F) The suppressive effects of population I CD161^−^, population III CD161^−^ and population III CD161^+^, Treg cells on (E) IFN-γ and (F) IL-2 production by autologous Teff cells were determined by supernatant cytokine concentrations (left) and percentage suppression (right) and expressed as mean + SD of two samples pooled from two independent experiments. (G) The suppressive function of population III CD161^+^ Treg cells after culture under IL-17-inducing conditions was determined by inhibition of CFSE dilution and is shown as a representative example (left) and mean +SD of two donors (right). (H) The concentration of IFN-γ (top left) and IL-2 (bottom left) and the percentage suppression of each cytokine (right) are shown as mean + SD of two samples pooled from two independent experiments. *p < 0.05, one-way ANOVA.

To further characterize the population of interest, FOXP3 expression was evaluated. Population III CD161^+^ Treg cells expressed less FOXP3 protein in comparison with population III CD161^−^ cells (Fig. 3A and B). Given the lower expression of FOXP3, and previous suggestions that population III is nonsuppressive [32], we tested their capacity to suppress proliferation of auto-logous CFSE-labeled Teff cells. We found that suppressive function in population III CD161^+^ Treg cells was similar to other Treg-cell populations (Fig. 3C and D). Likewise, their ability to suppress production of IFN-γ and IL-2 production from Teff cells was comparable to those of other Treg subpopulations (Fig. 3E and F). These data demonstrate that population III CD161^+^ cells have regulatory properties and further confirmed that these are not contaminating Teff cells of the Th17 or Th1 lineage.

To determine whether these cells lose their suppressive function upon IL-17 induction, population III CD161^+^ Treg cells were cultured under IL-17-inducing conditions for 5 days, then washed and co-cultured with autologous CFSE-labeled Teff cells in standard suppression assays. Not only did the Treg cells retain suppressive function (Fig. 3F and G), but their suppressive capacity was also enhanced following exposure to IL-17-inducing conditions (mean ± SD percentage suppression 56.8 ± 14.5% versus 92.0 ± 1.3% pre- and post-IL-17 induction, respectively; *p* < 0.05). Similarly, these Treg cells potently regulated IFN-γ and IL-2 production from Teff cells (Fig. 3H) after they had been exposed to IL-17-inducing conditions. These data suggest that IL-17 production is compatible with potent suppressive function in vitro.

### Population III CD161^+^ Treg cells are phenotypically similar to other Treg-cell subpopulations

**Figure 4:**
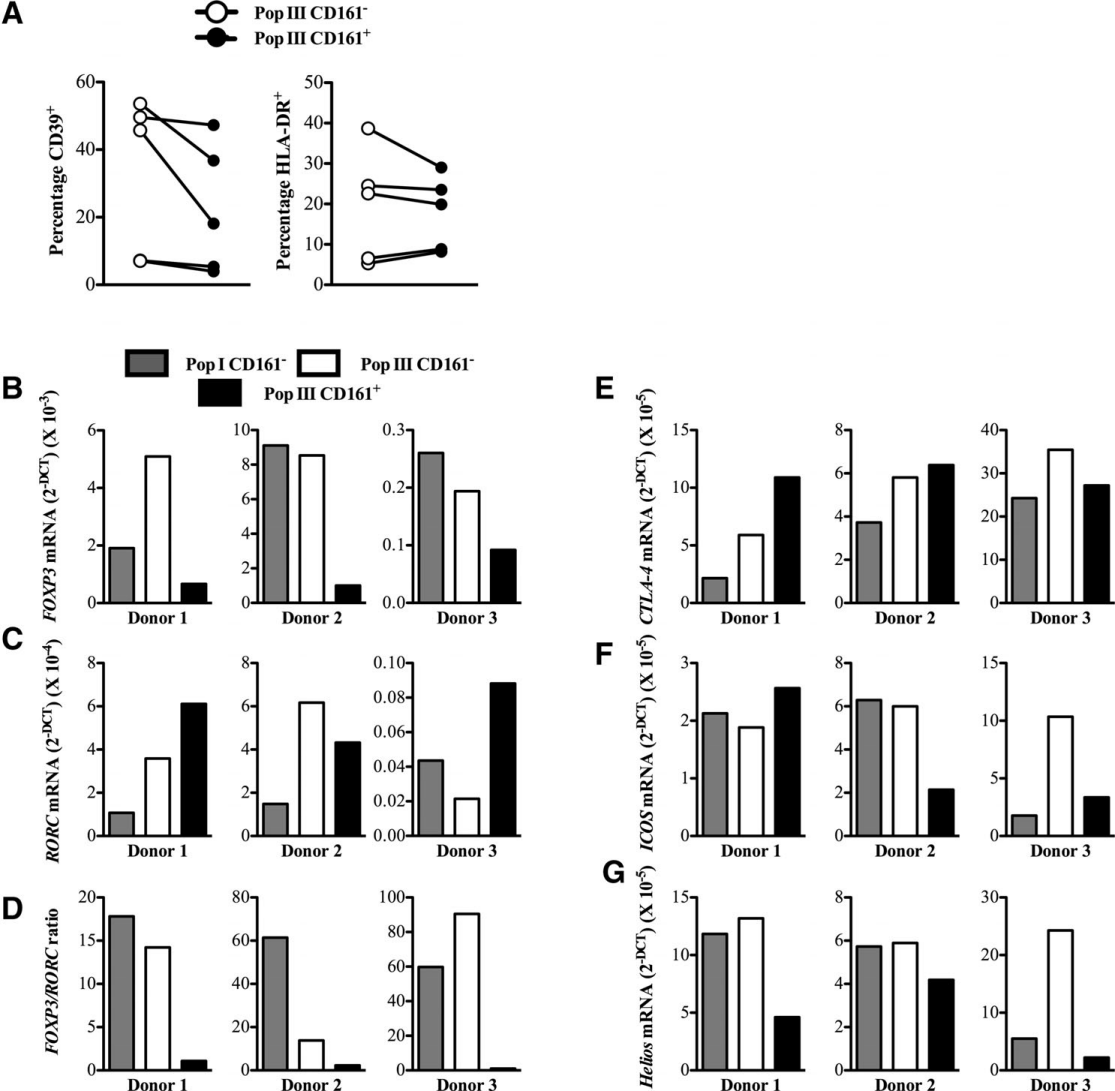
Immunophenotyping of freshly isolated population III CD161+ Treg cells. (A) The percentages of Treg cells staining positive for CD39 (left) and HLA-DR (right) in population III CD161^−^ and CD161^+^ Treg cells is shown. Paired data from five independent experiments are shown. (B–G) Gene expression profiling of freshly isolated population I CD161^−^, population III CD161^−^, and population III CD161^+^ Treg cells for (B) *FOXP3*, (C) *RORC*, (D) *FOXP3/RORC* ratio, (E) *CTLA-4*, (F) *ICOS*, and (G) *Helios* is shown. Individual data from three independent healthy donors are shown.

Population III CD161^+^ Treg cells were further characterized to determine how similar they are to other Treg-cell subpopulations at baseline. We first examined expression of CD39, an ecto-enzyme involved in Treg-cell function [38], and HLA-DR, a marker of Treg cells with early contact-dependent suppression [39], by flow cytometry, comparing CD161^−^ with CD161^+^ Treg cells in population III and found no significant differences between the percentages of Treg cells that express these markers on the two subpopulations, although there was a tendency to lower expression of CD39 (Fig. 4A). Likewise, we compared expression of *FOXP3*, *RORC*, *CTLA-4* (essential for Treg-cell function [40]), *ICOS* (Inducible T-cell co-stimulator; ICOS^+^ Treg cells suppress DCs via IL-10 and TGF-β [41]), and *Helios* (a putative marker of thymically derived Treg cells [42]) by qRT-PCR on populations I CD161^−^, III CD161^−^, and III CD161^+^ Treg cells obtained from freshly sorted healthy donor Treg cells (Fig. 4B–G). As noted above, population III CD161^+^ Treg cells express less *FOXP3* than the other populations (Fig. 4B) and variable basal levels of *RORC* with a tendency for higher expression in CD161^+^ cells (Fig. 4C), resulting in a quantitative *FOXP3*/*RORC* mRNA ratio in population III CD161^+^ Treg cells that favors *RORC*, compared with that of the other two populations (Fig. 4D).

At baseline, population III CD161^+^ Treg cells expressed similar amounts of *CTLA-4* (Fig. 4E) but lower quantities of *Helios* (Fig. 4G) than population III CD161^−^ Treg cells, suggesting a difference in these two populations either in their origin or context of generation. Consistent with this notion, analysis of “naïve” Treg cells from fresh human umbilical cord blood-derived CD4^+^ T cells, identified few population III CD161^+^ Treg cells, which produced only minimal amounts of IL-17 (Supporting Information Fig. 6). *ICOS* expression was more variable, being lower in two out of three donors in population III CD161^+^ Treg cells (Fig. 4F).

Overall, these data indicate that CD161-expressing Treg cells are not only functionally suppressive, but also possess phenotypic molecular characteristics that enable them to further differentiate to Th17 cells upon IL-1β stimulation.

### Population III CD161^+^ Treg cells are increased within inflamed joints

**Figure 5:**
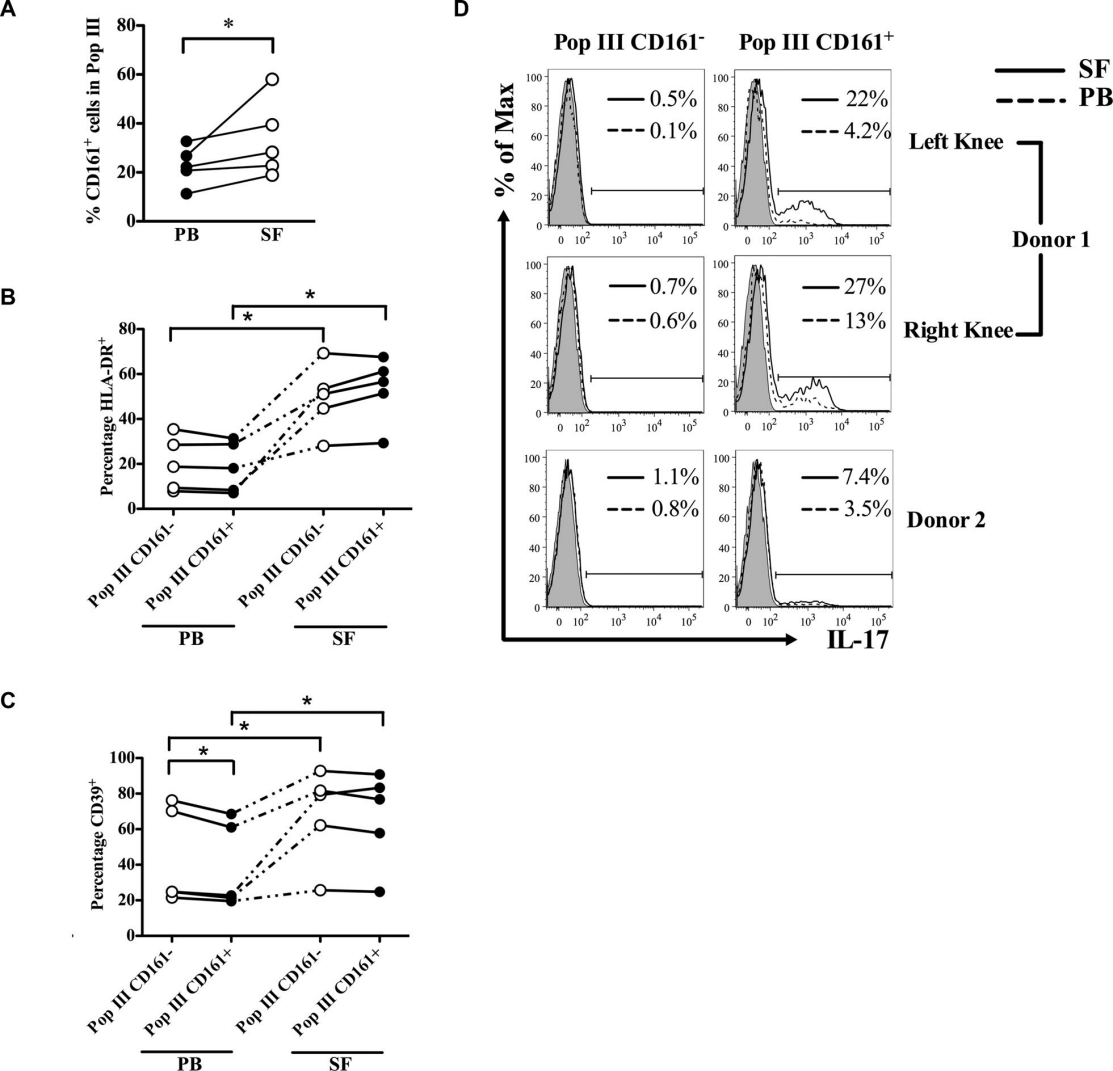
Characteristics of population III Treg cells in PB and SF from patients with inflammatory arthritis. (A) The percentage of CD161^+^ cells in population III Treg cells is shown. (B, C) The percentages of HLA-DR^+^ and CD39^+^ cells in population III CD161^−^ (open symbols) and population III CD161^+^ (filled symbols) Treg cells in PB and SF. Samples and cells from the same patients are connected with a dotted or solid line. (D) IL-17 production by paired population III CD161^−^ (left) and CD161^+^ (right) Treg cells in SF (solid line histograms) and PB (dashed line histograms) of two donors with inflammatory arthritis is shown. Numbers in each histogram show percentage positive for IL-17 in SF (top) and PB (bottom). *p < 0.05, paired *t*-test.

Having identified the human Treg-cell subpopulation with IL-17 potential, and established that this cell type is phenotypically similar to other human Treg cells within population III, we next sought to determine whether these cells are present within actively inflamed human environments. We therefore enumerated population III CD161^+^ Treg cells from paired PB and synovial fluid (SF) (of inflamed knee) samples of five patients with inflammatory arthritis (*n* = 1 rheumatoid arthritis, *n* = 4 psoriatic arthritis; Table 1) by flow cytometry (Fig. 5).

**Table 1:** Clinical characteristics of patients with inflammatory arthritis

Age	Sex	Pathology	Disease duration (years)	RhF	Erosive	Medication	DAS28	ESR	CRP
74	M	RA	5	+	Y	MTX	4.23	18	18
51	M	PsA	25	−	N	NSAID	4.3	20	17
72	M	PsA	13	+	Y	Etanercept	3.5	N/A	140
34	M	PsA	11	−	N	Etanercept	3.98	83	141
37	F	PsA	6	−	N	MTX	4.54	80	32

CRP: C-reactive protein; DAS28: disease activity score using 28 joint counts; ESR: erythrocyte sedimentation rate; MTX: methotrexate; N/A: not available; NSAID: nonsteroidal anti-inflammatory drugs; PsA: psoriatic arthritis; RA: rheumatoid arthritis; RhF: rheumatoid factor.

The percentage of CD161^+^ cells within population III Treg cells was significantly higher in SF than PB (Fig. 5A), suggesting that there is an accumulation of Treg cells with IL-17 potential at inflamed sites relative to PB. When we studied expression of HLA-DR and CD39 on Treg cells from these sites, we identified a greater percentage of HLA-DR expressing cells in population III Treg cells, whether CD161^−^ or CD161^+^, in the joint fluid compared to PB (Fig. 5B), indicating that these cells were recently activated, as previously suggested [43]. Similar to the pattern observed in healthy donors (Fig. 4A), a slightly lower percentage of CD39-expressing cells were found in PB population III CD161^+^ Treg cells compared with their CD161^−^ counterparts (Fig. 5C). In the SF, the percentage of CD39^+^ cells was increased in all population III CD161^−^ Treg cells and in all CD161^+^ Treg cells compared to their blood counterparts (Fig. 5B). To determine which Treg-cell subpopulation produces IL-17 in the inflammatory environment, we stained paired PB and SF mononuclear cells for IL-17 and gated on subpopulations of Treg cells. In both PB and SF, although the majority of IL-17 was produced by non-Treg cells (Treg cells accounted for 15 ± 1.41% of IL-17^+^ cells), IL-17 production from Treg cells was restricted to CD161^+^ population III (Fig. 5D), with SF Treg cells producing more IL-17 than their paired PB counterparts. Taken together, these data suggest that CD161^+^ Treg cells with the potential for IL-17 induction accumulate at sites of inflammation and are the principal source of IL-17 from Treg cells.

## Discussion

Here, we set out to identify the human Treg subpopulation that can be induced to produce IL-17 and to characterize its phenotypic features. We confirm that a proportion of the human Treg-cell pool in circulation (approximately 5–10% of total Treg cells) can be induced in vitro to produce IL-17 when activated in the presence of IL-1β, in a STAT3-dependent manner. We further demonstrate that “IL-17 potential” is isolated to population III (CD4^+^CD25^++^CD127^lo^CD45RA^−^) Treg cells and that this effect is almost exclusively restricted to a subpopulation within population III that expresses the NK-cell marker CD161. Opposed to a previous key publication [32], we find that at baseline these cells are functionally as suppressive and have similar phenotypic/molecular characteristics to subpopulations I and II of Treg cells, but that they have the potential to produce IL-17 while maintaining their suppressive function. Finally, we show that Treg cells with population III phenotypic features accumulate at sites of inflammation and that these cells are the main source of IL-17 from Treg cells at those sites.

CD4^+^CD25^+^ Treg cells in inflammatory arthritis have been studied extensively over the past years. Although conflicting data exist regarding Treg-cell frequencies and function in PB of inflammatory arthritis patients, studies generally agree on an increase in Treg-cell frequencies in the SF, that are functionally suppressive ex vivo, sometimes at an enhanced level [44–47]. The inflammatory environment of the arthritic joint may, however, interfere with Treg-cell-mediated suppression either by inducing resistance to suppression in Teff cells or by subverting Treg-cell function [48]. Our demonstration that a distinct population of CD4^+^CD25^++^CD127^lo^CD45RA^−^ Treg cells expressing CD161 is increased at the site of inflammation in inflammatory arthritis and that IL-17 production from Treg cells is restricted to these, suggests that these cells may be prone to producing IL-17 in the presence of IL-1β, which is present at increased levels in the inflamed arthritic joint. It remains to be determined, however, whether such an induction occurs in vivo. Our in vitro data indicate that CD161^+^ population III Treg cells in a noninflammatory environment are fully suppressive and phenotypically express similar markers to other Treg cells, in particular CD39, HLA-DR, and CTLA-4. In addition, after culture in IL-17-inducing conditions in vitro, these cells not only retain their suppressive function but demonstrate enhanced regulatory properties, consistent with those seen in Treg cells from inflamed SF. Using an in vitro model, we recently showed that activated monocytes, which are abundantly present at sites of inflammation and are potent producers of IL-1β, increase the percentage of IL-17^+^ cells in human CD4^+^CD25^+^CD127^lo^CD45RO^+^ Treg cells; however, these Treg cells maintained their ability to suppress effector T-cell proliferation concurrently with IL-17 production [49]. This is in line with recent findings that IL-17 production does not equivocally define a pro-inflammatory and destructive phenotype but can also contribute to tissue repair and integrity [50].

Of particular interest is FOXP3 expression in population III CD161^+^ Treg cells. Of the three subpopulations, we studied (populations I CD161^−^, III CD161^−^, and III CD161^+^), population III CD161^+^ expressed the least *FOXP3* mRNA and FOXP3 protein at baseline. Coupled to this, *RORC* levels were higher in the CD161^+^ population resulting in a *FOXP3*/*RORC* ratio that was the lowest of the three populations, making these cells appear poised for IL-17 production. As FOXP3 physically interacts with and inhibits RORC and RORA, preventing them from binding their genomic targets [51], reduced FOXP3 expression in plastic Treg-cell subpopulations could, in addition, be permissive for activation of the RORC transcriptional programme.

In contrast to our observations that population III Treg cells are highly suppressive, Miyara et al. [32] described population III Treg cells as nonregulatory. This discrepancy may be related to differences in Teff-cell populations and stimulation conditions selected in suppression assays. Likewise, murine fate-mapping models that demonstrate limited in vivo Treg-cell plasticity [28], although elegant, have sufficient limitations [30] that they do not provide definitive answers to the question of Treg-cell plasticity and do not explain why T cells with the characteristics we describe in this manuscript have also been identified at inflamed, but not healthy, areas in the bowel of patients with Crohn's disease [29].

Mechanistically, both IL-1β and STAT3 signaling appears critical for induction of IL-17 in human Treg cells. Although the requirement for STAT3 activation for Th17-cell differentiation in conventional T cells is well established [34], it is likely that STAT3 activation in Treg cells occurs through nonconventional means, as described previously in nonimmune cells [52], because the IL-1R does not directly interact with JAK proteins. This may also explain why classical STAT3 activators, such as IL-6, do not appear to play a role in IL-17 induction in human Treg cells in this or other studies [25,26]. IL-2 has previously been shown to antagonize conventional Th17 differentiation in murine cells [33], so the synergistic effect of IL-2 with IL-1β to promote IL-17 induction in human Treg cells appears unexpected. However, in human cells, the common γ chain (γc) using cytokines, including IL-2, have been shown to potently activate Th17 signature cytokines in specific CD4^+^ memory T cells [53] and, therefore, may stimulate CD161^+^ Treg cells in a similar manner. We believe that one key role of IL-2 in IL-17 induction from Treg cells is to upregulate IL-1R1, as has also been demonstrated by others [12], sensitizing Treg cells to IL-1 signaling.

These observations suggest that Treg cells are not terminally differentiated and can alter their phenotype as dictated by their local cytokine microenvironment. That some, but not all Treg cells, have the capacity to express programs of other lineages is important, as universal potential to undergo fate switching to pro-inflammatory lineages in Treg cells at the site of inflammation could represent a greater risk for the development of auto-immune diseases than is seen during the normal human response to infectious challenges. In the context of Treg-cell-based therapy for induction of tolerance to transplanted organs or production of remission in autoimmune diseases, transfer of a Treg-cell product that contains a subpopulation that can express pro-inflammatory cytokines could potentially be damaging. However, our data suggests that IL-17 production from a Treg-cell subpopulation with “IL-17 potential” is not necessarily accompanied by a loss of regulatory function, so exclusion of these cells from the cell product for Treg-cell therapy, for example by CD161 depletion, may not be necessary. This assertion is supported by Treg-cell-based clinical trials in the context of bone marrow transplantation [16] and type 1 diabetes mellitus [54], in which suggestive adverse effects have not been reported.

## Materials and methods

### T-cell separation, FACS sorting, and flow cytometry

CD25^+^ and CD25^−^ CD4^+^ T cells were separated from human buffy coats as previously described [55]. Baseline FOXP3 and IL-17 expression is shown in Supporting Information Figure 2A. Flow cytometry and FACS sorting (routinely to a purity ≥95%), using CD4^+^ T cells stained for CD4-Qdot605, CD161-allophycocyanin, CD127-PerCP-Cy5.5, CD39-FITC, HLA-DR-EF450, CCR6-FITC, IL-1R1-CFS (all eBioscience), CD25-PE (BD), and CD45RA-AF700 (Biolegend) was carried out using an LSR II and FACSAria (both BD), respectively. Appropriate isotype controls and Fluorescence Minus One (FMO) controls were used to assign gates. ICS for FOXP3 was carried out using the kit from eBioscience according to manufacturer's instructions. For co-staining of cytokines and transcription factors, PMA (50 ng/mL), ionomycin (1 mM) (both Sigma), and Brefeldin A (3 μg/mL; eBioscience) were added to cell cultures 4.5 h before ICS with FOXP3, IL-17, and/or RORC (all eBioscience) as required. All patient samples were collected following informed written consent and studies were approved by appropriate ethics committees.

### Cell culture and suppression assays

Cells were cultured in complete medium as previously described [56] and polyclonally activated with anti-CD3/CD28 microbeads (Invitrogen) in the presence or absence of 10 ng/mL IL-1β (R&D Systems), 10 IU/mL IL-2 (Roche), or 10 ng/mL IL-1β + 10 IU/mL IL-2. Suppression of Teff-cell proliferation by Treg cells was assessed using the standard CFSE dilution method [57] comparing Teff cells cultured alone to those co-cultured 1:1 with Treg cells.

### Cytokine measurement

Sandwich ELISA for human IL-17 (R&D systems) in T-cell supernatants was carried out in duplicate according to manufacturer's instructions. Cytokine concentrations were interpolated from contemporaneously acquired standard curves.

### Western blotting

Whole cell extracts were prepared by cell lysis in Laemmli sample buffer. Proteins were resolved by SDS-PAGE on 8% Tris-Glycine gels (Invitrogen) and electrotransferred onto polyvinylidene fluoride membranes (Immobilon), Immunoblotting was performed according to standard protocols, with overnight incubation in primary antibodies: anti-pY-STAT3 Tyr 705, anti-PY-STAT5 (Cell Signaling, New England Biolabs), pan-STAT5 (BD Biosciences), or anti-STAT3 (SantaCruz Biotechnology) and 1-h incubation in appropriate secondary antibodies. Bound antibodies were detected using ECL-Plus reagents (Pierce).

### RNA extraction and qRT-PCR

RNA extraction (RNAqueous micro extraction kit (Ambion, Applied Biosystems, CA, USA)) was followed by cDNA synthesis using a Verso™ cDNA kit (Thermo Scientific). qRT-PCR was performed in triplicate using the ABI Prism 7900 HT Sequence Detection System (SDS) and TaqMan gene expression kit (both Applied Biosystems). Gene-specific primers (all from Applied Biosystems) are shown in Supporting Information Table 1. Average CT values were exported using SDS 2.3 software and normalized against the control probe 18S using the formula 2∧^[CT (18s)-CT (S)]^ where S = sample.

### Data analysis

Data analysis used Microsoft Excel and GraphPad Prism 5 (GraphPad software, USA). Statistical analysis was performed by using paired parametric and nonparametric tests as appropriate. Multiple datasets were compared by repeated measures ANOVA (Tukey's posthoc test) and Friedman tests (Dunns posthoc test) for parametric and nonparametric data, respectively. A *p* value < 0.05 was considered statistically significant.

## Abbreviations

ICS: intracellular staining
PB: peripheral blood
SF: synovial fluid
Teff: effector T

## Supporting Information

As a service to our authors and readers, this journal provides supporting information supplied by the authors. Such materials are peer reviewed and may be re-organized for online delivery, but are not copy-edited or typeset. Technical support issues arising from supporting information (other than missing files) should be addressed to the authors.

Figure S1. IL-1R1 is upregulated by IL-2 on Tregs. Figure S2. FOXP3 and IL-17 staining Figure S3. Intracellular staining for pY-STAT3 and pY-STAT5 in Tregs. Figure S4. Gating strategy for identification of Treg subpopulations I, II and III Figure S5. CCR6 and IL-1R1 expression on Tregs from population III. Figure S6. Cord Blood Tregs have few CD161+ Tregs in population III and produce almost no IL-17. Table S1. Primers used for qRT-PCR
