# Supplementary material for: CD161 expression characterizes a subpopulation of human regulatory T cells that produces IL-17 in a STAT3-dependent manner
Source: Eur J Immunol. 2013 May 15;43(8):2043–54. doi: 10.1002/eji.201243296 (PMC3815561; doi:10.1002/eji.201243296)
Supplement: Supplementary file 1 [file eji0043-2043-sd1.pdf]

European Journal of Immunology

Supporting Information
for

DOI 10.1002/eji.201243296

Behdad Afzali, Peter J. Mitchell, Francis C. Edozie, Giovanni A.M. Povoleri, Sophie E. Dowson, Laura Demandt, Gina Walter, James B. Canavan, Cristiano Scotta, Bina Menon, Prabhjoat S. Chana, Wafa Khamri, Shahram Y. Kordasti, Susanne Heck, Bodo Grimbacher, Timothy Tree, Andrew P. Cope, Leonie S. Taams, Robert I. Lechler, Susan John and Giovanna Lombardi

CD161 expression characterizes a subpopulation of human regulatory T cells that produces IL-17 in a STAT3-dependent manner

© 2013 The Authors. *European Journal of Immunology* published by Wiley-VCH Verlag GmbH & Co. KGaA Weinheim.

Supplementary Table 1. Primers used for qRT-PCR

GENE	SEQUENCE
FOXP3	Hs01085834_m1*
CTLA-4	Hs03044418_m1*
RORC	Hs01076112_m1*
Helios	Hs00212361_m1*
ICOS	Hs00359999_m1*
IRF-4	Hs01056533_m1*
18S	Hs03003631_g1

All probes are from Applied Biosystems, California.

Supporting Information Figure 1. IL-1R1 is upregulated by IL-2 on Tregs.

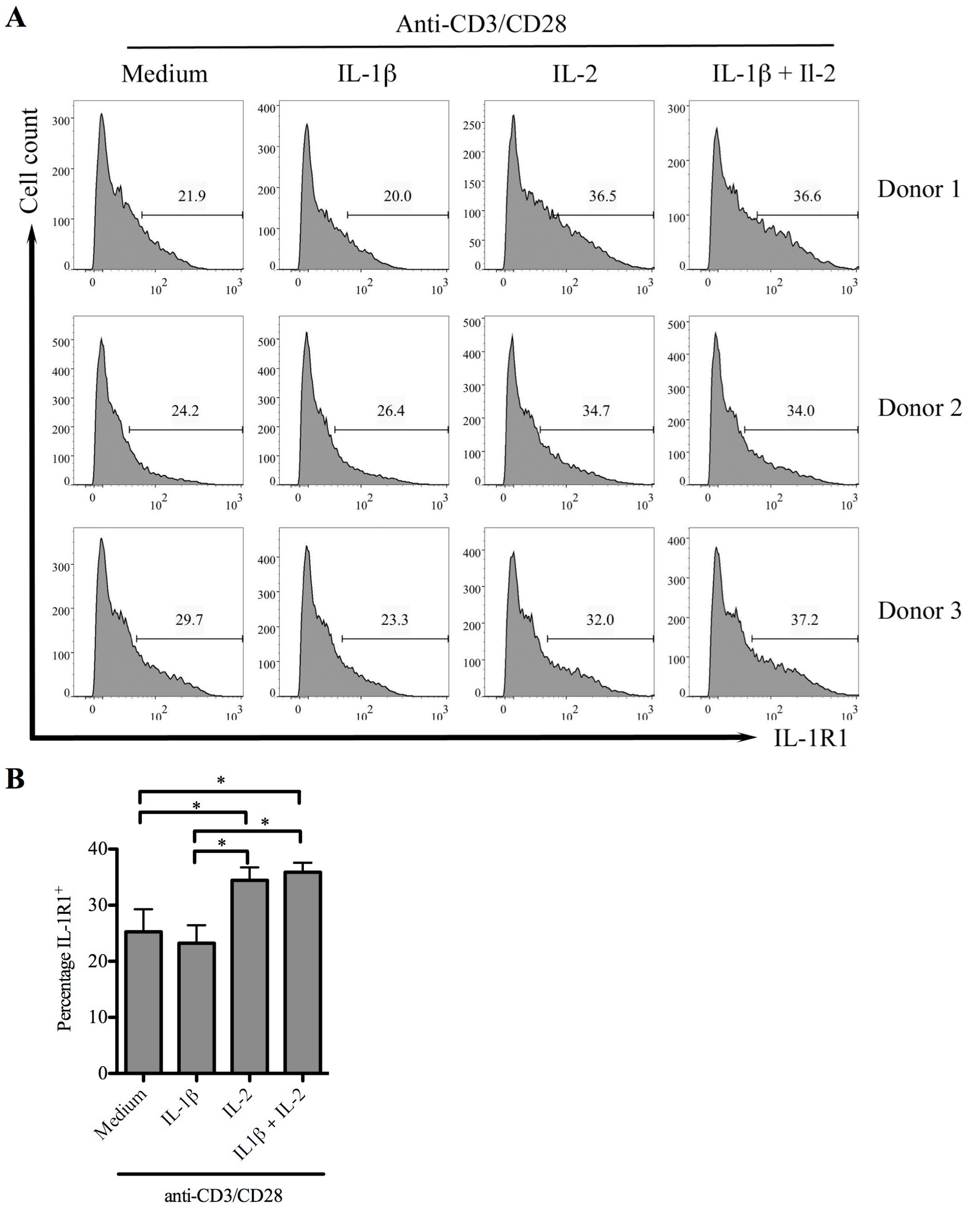

Freshly isolated Tregs were activated *in vitro* with anti-CD3/CD28 coated microbeads in the presence and absence of IL-1β, IL-2 and IL-1β plus IL-2. After three days, cells were stained for surface expression of IL-1R1. **A and B**, individual histograms (**A**) and cumulative data (mean ± s.d.) (**B**) of IL-1R1 expression from three independent donors. *$p<0.05$.

Supporting Information Figure 2. FOXP3 and IL-17 staining

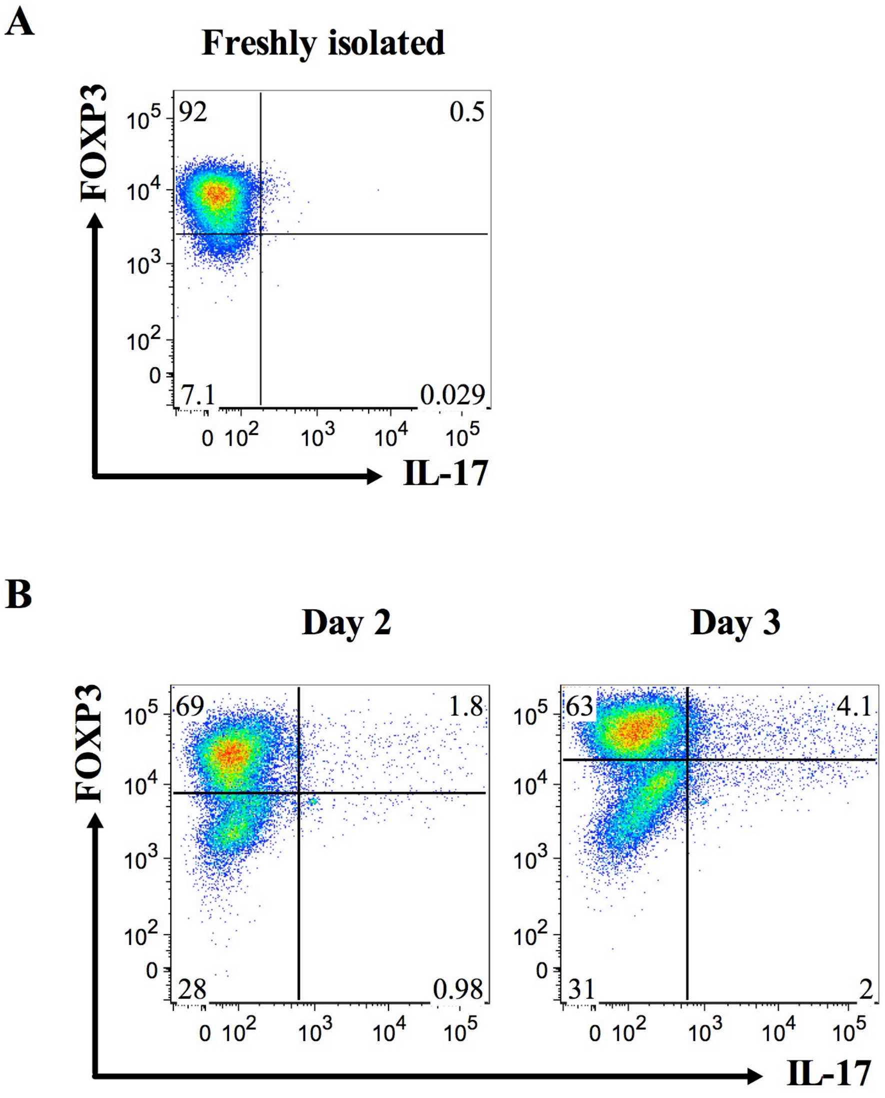

Freshly isolated CD4^{+}CD25^{+} Tregs were stained for FOXP3 and IL-17 at baseline (immediately after isolation) (**A**) and 2 and 3 days (**B**) after activation *in vitro* with anti-CD3/CD28 + IL-1β + IL-2. Dot plots show representative figures from 2 independent experiments.

Supporting Information Figure 3. Intracellular staining for pY-STAT3 and pY-STAT5 in Tregs.

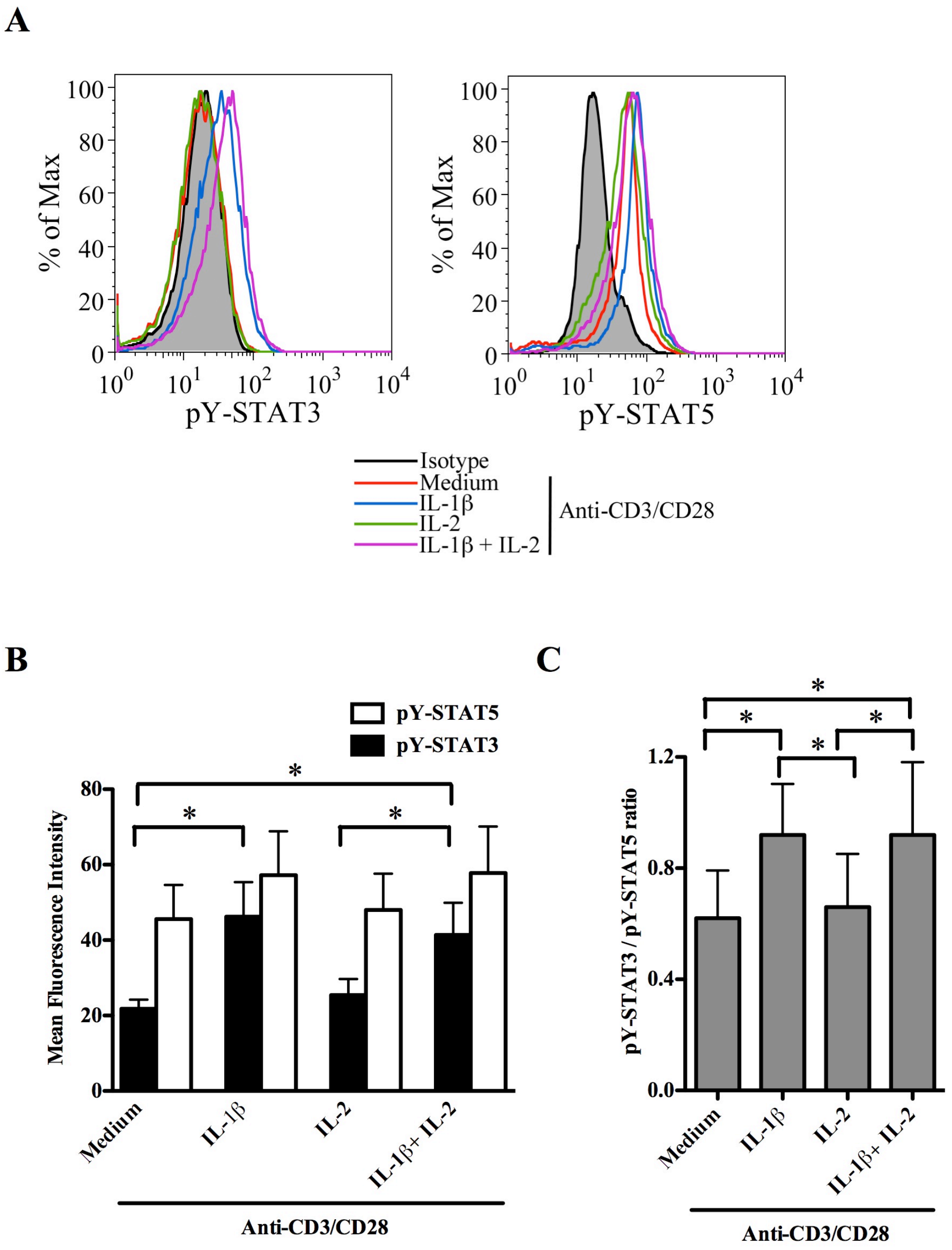

Tregs were activated with anti-CD3/CD28 coated microbeads in the presence or absence of IL-1β, IL-2 and IL-1β plus IL-2 for 5 days, followed by ICS for pY-STAT3 and pY-STAT5 to confirm Western blot data on a *per cell* basis. **A,** a representative example of pY-STAT3 (left panel) and pY-STAT5 (right panel) staining from 5 independent experiments. Isotype is shown in grey. **B,** mean fluorescence intensity (MFI) of pY-STAT3 and pY-STAT5 staining, showing cumulative data (mean ± s.d.) from 5 independent experiments. **C,** ratio of pY-STAT3 to pY-STAT5 MFI, showing cumulative data (mean ± s.d.) from 5 independent experiments. *$p<0.05$

Supporting Information Figure 4. Gating strategy for identification of Treg sub-populations I, II and III

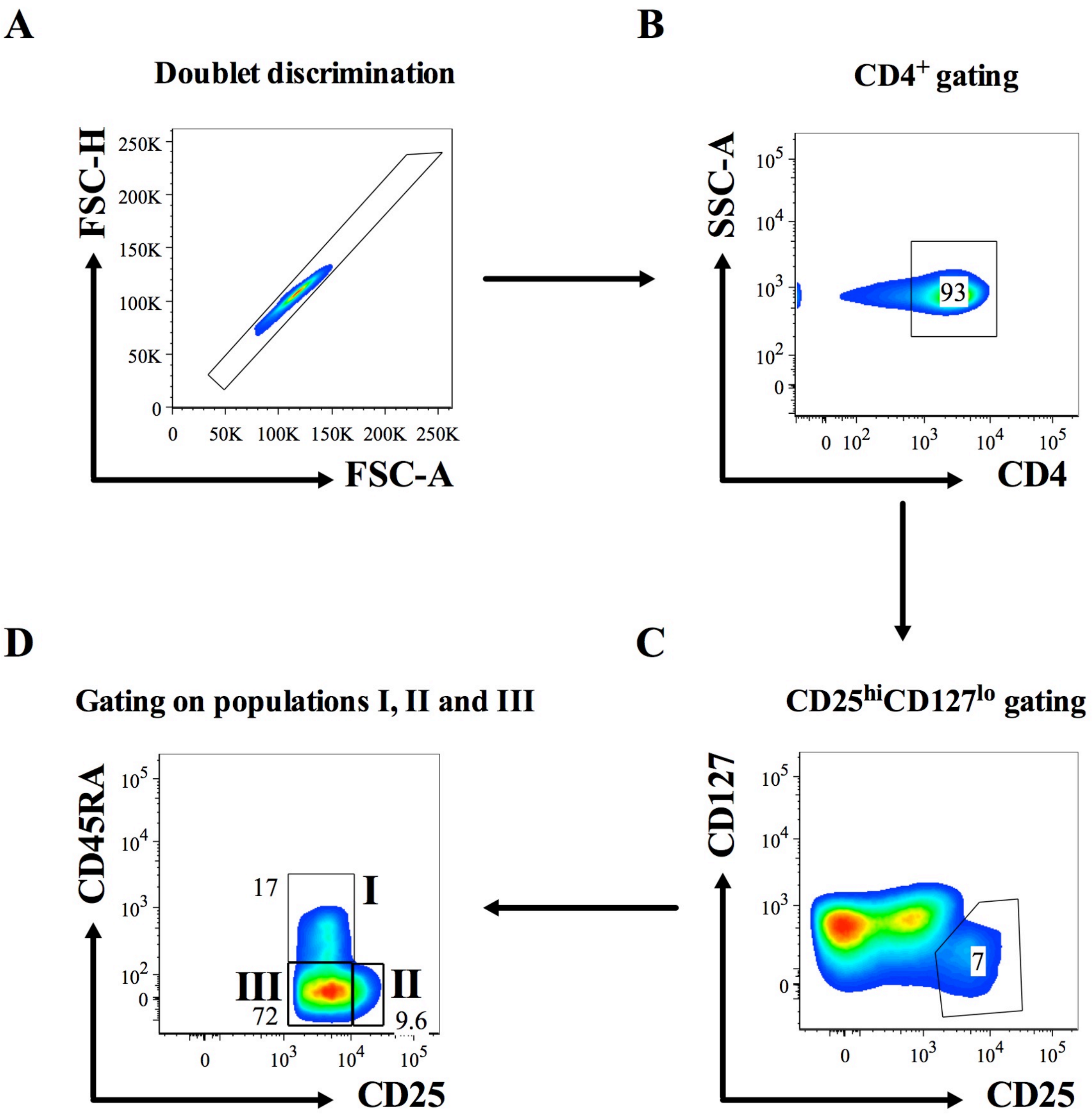

Gated lymphocytes were analysed first by doublet discrimination (**A**), then $CD4^+$ selection (**B**), followed by gating $CD25^{hi}CD127^{lo}$ cells (**C**). Subsequently, populations I, II and III were gated based on expression of CD25 and CD45RA as shown (**D**)

Supporting Information Figure 5. CCR6 and IL-1R1 expression on Tregs from population III.

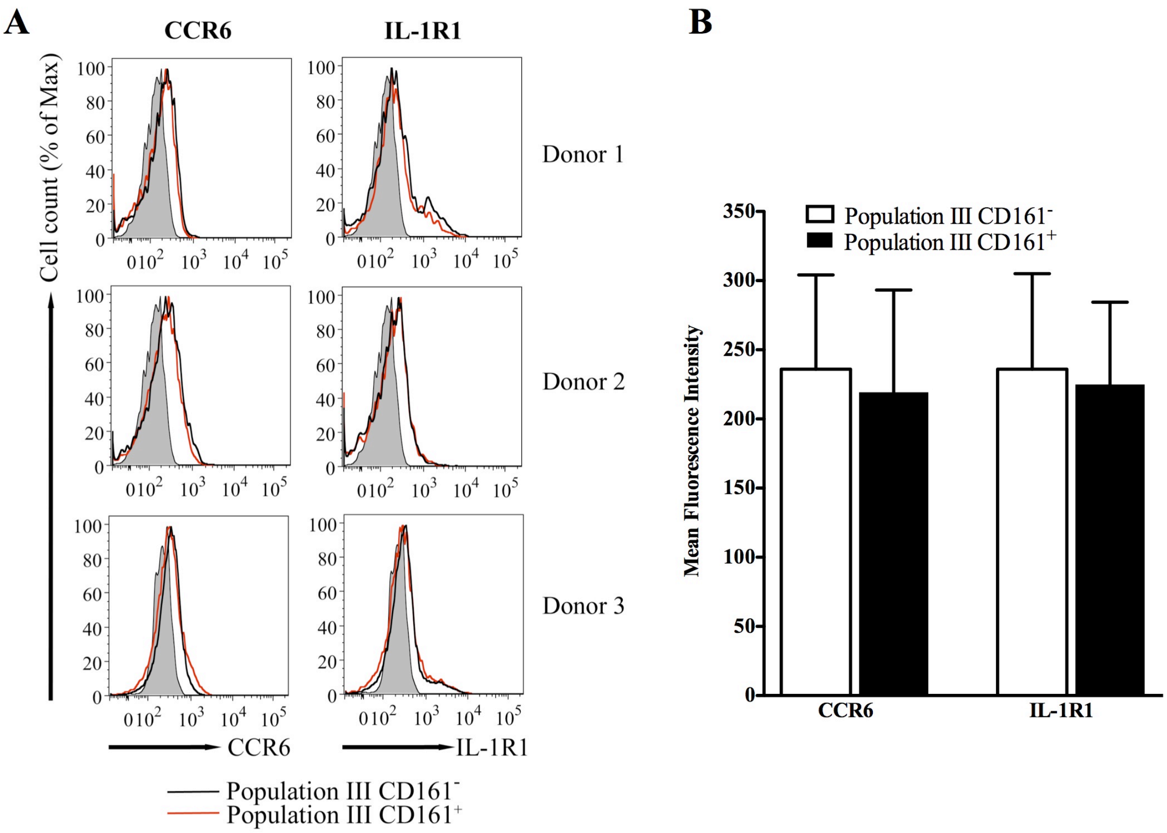

A, histograms showing expression of CCR6 (left panels) and IL-1R1 on freshly isolated population III $CD161^{+}$ and population III $CD161^{-}$ Tregs from 3 independent donors. Gray histograms show isotypes. **B,** mean fluorescence intensity (MFI) of CCR6 and IL-1R1 on population III $CD161^{+}$ and population III $CD161^{-}$ Tregs, showing cumulative data (mean ± s.d.) from the 3 donors in A.

Supporting Information Figure 6. Cord Blood Tregs have few CD161$^+$ Tregs in population III and produce almost no IL-17.

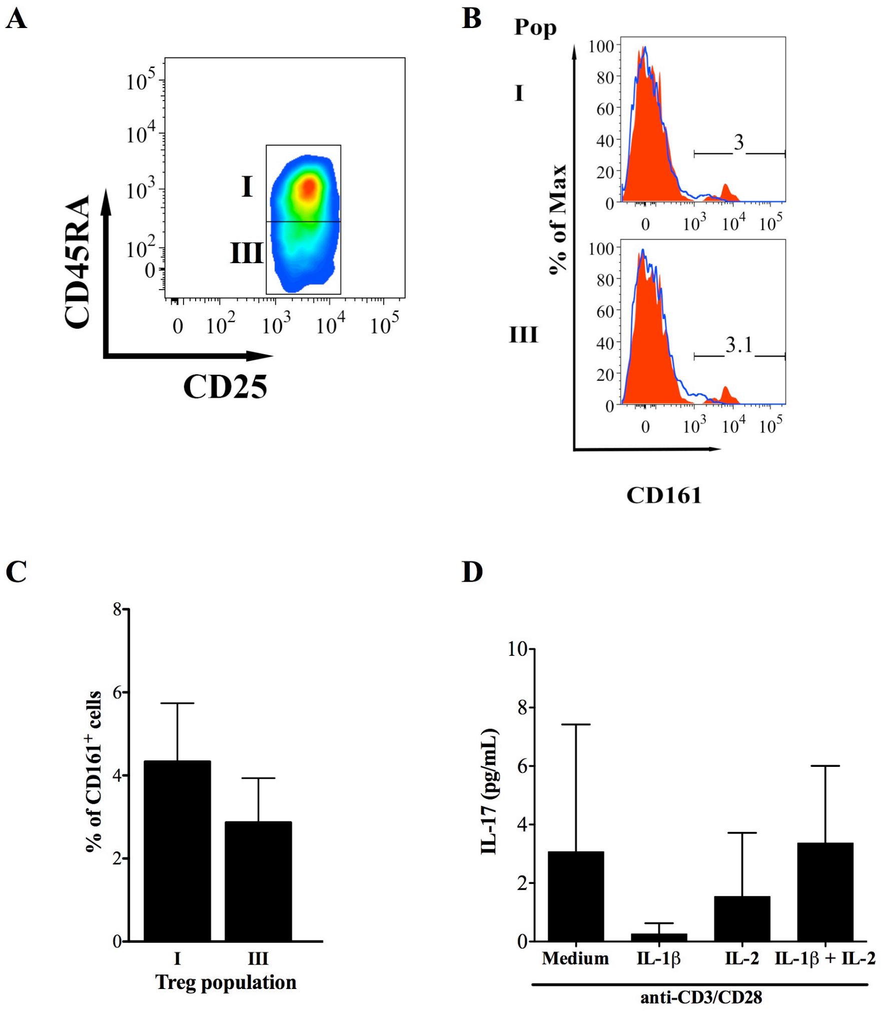

Cord blood Tregs were examined to determine the frequency of population III CD161$^+$ Tregs and ability to produce IL-17. **A,** representative dot plot, from 3 independent cord bloods examined, showing that cord blood Tregs contain population III but not population II. **B** and **C,** representative example (**B**) and pooled mean ± s.d. (**C**) CD161 expression on populations I and III Tregs from 3 independent cord blood samples examined. **D,** IL-17 production from "whole" cord blood Tregs activated in vitro in the absence (medium) and absence of IL-1β, IL-2, and IL-1β plus IL-2.
